# Fundamentals of Green Steel Production: On the Role of Gas Pressure During Hydrogen Reduction of Iron Ores

**DOI:** 10.1007/s11837-023-05829-z

**Published:** 2023-05-01

**Authors:** I. R. Souza Filho, Y. Ma, D. Raabe, H. Springer

**Affiliations:** 1 Max-Planck-Institut Für Eisenforschung, 40237 Düsseldorf, Germany; 2 Metallic Composites, RWTH Aachen University, 52072 Aachen, Germany

## Abstract

Out of the multitude of researched processing routes for sustainable ironmaking, hydrogen-based direct reduction and hydrogen plasma smelting reduction (HyPSR) are currently the most promising candidates for a successful industrial application. Both processes operate under gaseous atmospheres, which turn the partial and absolute pressure of hydrogen into a relevant process parameter. Here, we present first insights into the influence of total pressure and concentration of hydrogen on the reduction of hematite, focusing on the more pressure-sensitive route (HyPSR). The effect of pressure on the dissociation of H_2_ molecules into metastable H atoms or H^+^ ions,- and the overall hydrogen utilization is discussed using a thermodynamic approach. Validation experiments were conducted to testify the practical feasibility of adjusting these parameters. A decrease in the total pressure of the system from 900 mbar to 450 mbar resulted in an improved hydrogen utilization, thus suggesting that a larger population of H atoms can exist in the plasma arcs ignited under 450 mbar. An increase in the hydrogen concentration to 20 vol.% lead to undesired evaporation, likely because of a parallel increase in plasma temperature. Possibilities and challenges for exploiting these pressure-related phenomena for the industrial production of green steel are outlined and discussed.

**Supplementary Information:**

The online version contains supplementary material available at 10.1007/s11837-023-05829-z.

## Introduction

**Fig. 1:**
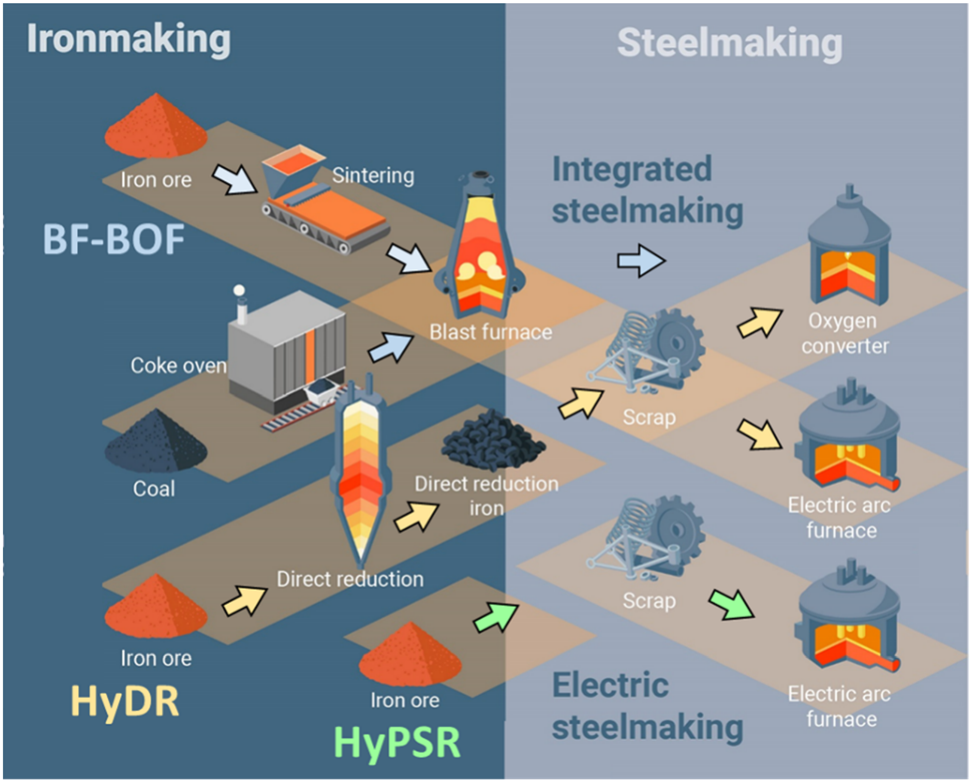
Different iron- and steelmaking routes. The blue arrows indicate the process path along the integrated blast furnace and basic oxygen furnace (BF-BOF) route. The yellow arrows show the sequence of the hydrogen-based direct reduction (HyDR) followed by melting in an electric arc furnace (EAF). The green arrows show the hydrogen plasma smelting reduction (HyPSR). Adapted from 10.1021/acs.chemrev.2c00799 (Color figure online).

Steel is the backbone of modern civilization and huge amounts are being produced worldwide—almost 2 billion tons in 2021 to facilitate economic growth and increasing standards of living conditions.^1^ Ever growing demand for extending infrastructure such as buildings and road networks especially in emerging economies render a circular steel economy (closed loop recycling) not yet feasible. Consequently, fresh iron has to be produced by the reduction of ores, at a current global fraction of nearly 2/3rd and only 1/3rd coming from melting scrap.^2^ Primary synthesis is currently achieved by utilizing fossil carbon (i.e., coal as the base for coke) to supply the required energy for both reduction and melting, as well as serve as a reducing agent of iron ores.^3^ The integrated processing route via blast- and basic oxygen furnace (BF-BOF, Fig. 1) is widely established.^4^ It covers about 70% of the current primary iron synthesis and has reached such a high level of efficiency that further thermodynamic improvement yields only asymptotic progress. Its reliance on carbon, however, means that huge amounts of CO_2_ are still being emitted, representing about 8% of the total global emissions.^5^

Decarbonizing the primary production of iron and steel is therefore one of the most powerful leverages for achieving the targeted emission goals to fight global warming.^5–7^ For that purpose, alternative energy sources and reduction agents are required. Transitory developments rely for example on natural gas instead of coke to reduce the CO_2_ footprint.^8^ For a truly green steel production though, the use of (electrical) energy from renewable sources is a must, which also has to be employed for supplying the reducing agent which is required to remove oxygen from iron oxide. While various types of compounds such as ammonia or certain toluols can be used as carriers for easier transport, hydrogen is the prime candidate as a reduction partner, as the reaction with iron oxides renders only water as a by-product.^8,9^ However, this radical change in reduction chemistry and energy source means that the production technology for steels, which has been developed and refined for over 3 millennia, needs to be completely reinvented, rebuilt, upscaled and rendered commercially viable at a 2 billion tons per year scale and all that within a few years’ time. This means that one of the most important global backbone industries (and the many downstream manufacturing sectors that depend on it) is now at the beginning of a disruptive transformation process, with many engineering solutions being explored, developed, upscaled and partially already tried and implemented at higher technology readiness levels (TRL). From the vast array of proposed and existing processing routes, two hydrogen reduction techniques are currently the most promising ones for potential widespread use, capable of coping with the immense tonnages demanded by the market (Fig. 1): (1) reduction of solid ore at elevated temperature in a flow of hydrogen gas (i.e., hydrogen-based direct reduction, HyDR), followed by melting of the reduced sponge iron,^9,10^ and (2) simultaneous melting and reducing ores in a modified electric arc furnace under a hydrogen-containing plasma (i.e., hydrogen plasma smelting reduction, HyPSR).^11–13^ The former route is similar to the existing MIDREX process (but without using natural gas) and on the brink of wider scale industrial application.^6,8,14^ The latter offers an even shorter process chain and higher efficiency, but is still on a lower technology readiness level. For both HyDR and HyPSR, many scientific questions need to be answered to better understand the underlying reaction mechanisms, increase efficiency especially in the consumption of costly hydrogen,^12^ and even discover possible new technological pathways for green steel production.^9^ These questions range from the influence of ore composition and microstructure on diffusion phenomena and phase transformations, over the best suitable type and composition of the gas atmosphere, to process temperature regimes and reactor design, to name but a few. The influence of gas pressure has not received considerable attention so far in ongoing research activities, although it can play a crucial role in chemical reactions. Here, we present first insights into the fundamental aspects of changing partial and absolute pressures—above and below standard conditions—in both hydrogen-containing gas and plasma atmospheres and explore challenges and potentials for this so far scarcely investigated parameter for green steel production.

### Effect of Gas Pressure on HyDR

Direct reduction technologies, such as shaft furnaces (e.g., MIDREX and HYL Energiron) and fluidized beds (e.g., FINMET and CIRCORED), have been developed and implemented at the industrial scale. These technologies currently deploy coal or natural gases as reductants. Therefore, HyDR could be quickly developed by modifying these mature technologies. The current version of the MIDREX process is operated slightly above ambient pressure (1.5–2.0 bar), while the HYL Energiron process is operated at a higher pressure above 5 bar.^15^ The operating pressure for the fluidized bed reactors is above 4 bar to ensure the fluidization of ore fines.^16^ For the HyDR processes, it is vital to understand the effect of gas pressure on the reaction behavior.

**Fig. 2:**
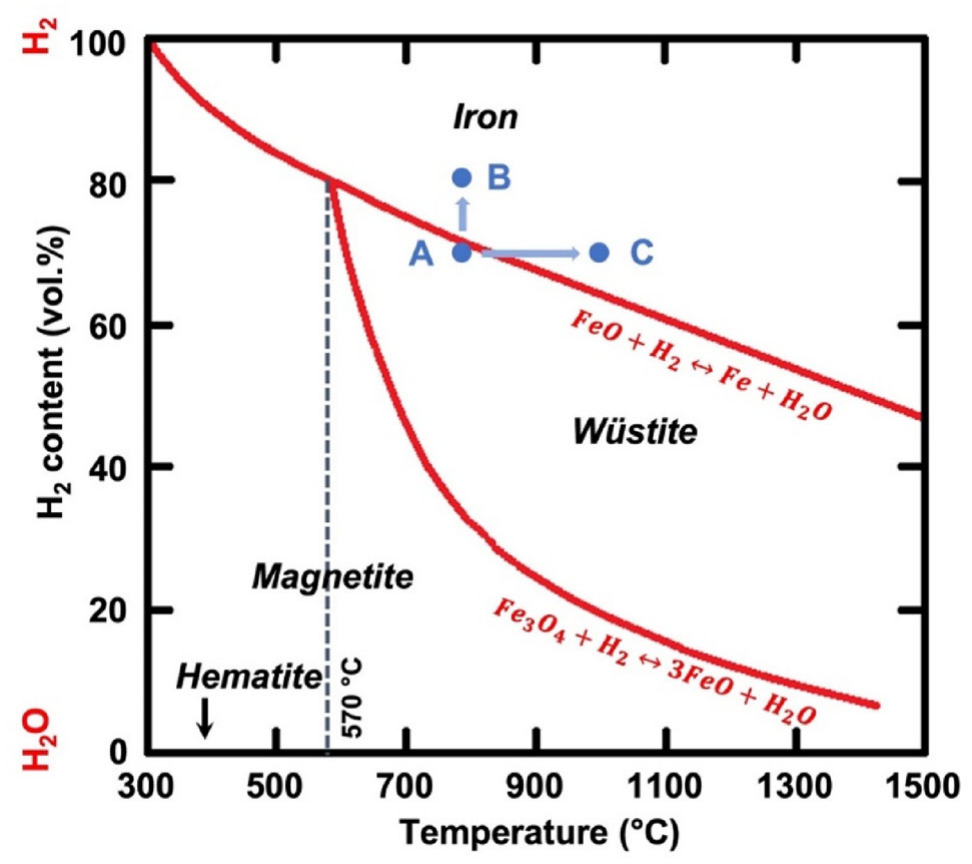
Schematic illustration of a Baur-Glässner diagram of the iron oxides-hydrogen system. Point A exemplifies the H_2_ partial pressure inside a shaft furnace where the reduction of wüstite into iron is thermodynamically not feasible at 800°C. To proceed with the reaction, either an increase in the H_2_ partial pressure to, e.g., point B or an increase in the temperature (e.g., to point C) is required.

A few studies have evaluated the effect of gas pressure on HyDR with pure reducing gases (H_2_ or CO) or gas mixtures, including H_2_, CO, H_2_O, CO_2_ and inert gas, etc.^17–20^ These studies observed the subtle effect of absolute pressure of gas mixture (in a range of 1–3 bar) on the reduction kinetics.^17,21^ In contrast, an increase in partial pressure of reducing gases (H_2_ or CO) effectively boosted the reduction kinetics.^19^ According to Le Chatelier’s principle, there should be no effect of absolute pressure on the reaction equilibrium when the moles of gases are equal on both sides of the reaction, such as the overall reaction for the reduction of iron oxides: Fe_2_O_3_ + 3H_2_ (or CO) → 2Fe + 3H_2_O (or CO_2_). However, the partial pressure of reducing gases (e.g., p_(H2)_/(p_(H2)_ + p_(H2O)_)) influences the driving force of the reaction,^9,21^ which plays a more important role than the absolute pressure. An increase in the partial pressure of reducing gases renders more moles of reducing gas within a confined volume of a reaction chamber. In such a case, the probability of gas molecule dissociation and of collisions of reducing gas molecule species with iron oxides increases significantly. When pure H_2_ was used, an increase in the absolute pressure (in a range of 5–36 bar) facilitated fast reduction kinetics.^18^ From a thermodynamic point of view, this effect can be understood as an increase in the partial pressure of H_2_, as the amount of hydrogen gas is significantly increased in the reactor (with a fixed volume), while the amount of H_2_O remains at a similar level. In a countercurrent-flow shaft furnace, the partial pressure of H_2_ drops quickly when water is produced by the reduction of wüstite to iron in the lower section of the shaft furnace. As the operation point approaches A, as shown in Fig. 2, this condition can impede the further reduction of wüstite. When the same amount of water is produced, yet a higher pressure of pure hydrogen is imposed in the system, resulting in a higher partial pressure of hydrogen. Thus, the operating point shifts, for example, to point B. At this operating point, the driving force for the chemical reaction is higher than that at point A, allowing for a higher metallization degree and productivity. Otherwise, a higher temperature is demanded to proceed with the reaction (e.g., point C).

Nevertheless, there is a lack of systematic investigations of the pressure effect on HyDR in the literature. As there are several rate-controlling parameters, such as gaseous diffusion, chemical reaction, solid-state diffusion, etc*.*, the pressure effect on individual reduction and reactor scenarios should be better understood and decoupled. Also, the second-order effect of pressure, for instance, on the microstructure evolution during HyDR, remains unclear. A better understanding of these aspects will facilitate the more efficient operation of the HyDR reactors by further enhancing the HyDR kinetics and gas utilization.

### Effects of Pressure on HyPSR

When considering the plasma atmospheres in HyPSR reactors, the role of total and H_2_ partial pressure is not only relevant for perspectives of the process, reactor and feedstock developments but is also indispensable to tune the kinetics and equilibrium aspects of the dissociation reaction of H_2_ molecules into metastable hydrogen plasma species (e.g., H_2_ → 2 H, at 2500°C).^22–25^ This motivates us to elucidate the first insights on the thermodynamic equilibrium of hydrogen species such as H_2_, H and H^+^ in gas mixtures of Ar-H_2_ with different molar fractions of H_2_ and at different absolute pressures. In this study, we performed validation experiments in which hematite pieces were reduced via HyPSR under different atmospheric conditions. The findings are compared with those reported in the literature. We propose a general picture of the impact of pressure-related process parameters on the reduction of hematite into iron through HyPSR, aiming to support the next process and science-based investigations to be conducted to scale up this alternative green ironmaking route.

## Materials and Methods

### Thermodynamic Simulations

Equilibrium calculations for gas mixtures containing H_2_ and Ar were conducted using ThermoCalc software coupled with the SSUB5 SGTE (Scientific Group Thermodata Europe) substances database to assess the molar fractions of different species, including H_2_ molecules, H atoms, H^+^ ions and electrons within the temperature interval ranging from 500 K to 30,000 K. These calculations were performed under different absolute pressures (50 mbar, 450 mbar and 900 mbar) and different H_2_ molar fractions (viz., 0.1, 0.2 and 0.9).

The efficiency in hydrogen utilization during the reduction of a liquid iron oxide (Fe-28.9 wt.%O) with H_2_ molecules and H atoms at 2000°C (under equilibrium) was also calculated using ThermoCalc. The liquid phase was described using the TCS Metal Oxide Solutions Database (TCOX10). The gas phase was described using the SSUB5 SGTE database. Metallic and oxide vapors were also included in the gas phase.

### Hydrogen Plasma Smelting Reduction of Hematite

Laboratory-grade hematite, whose chemical composition is shown in Table I, was reduced via hydrogen-containing plasma atmospheres ignited in an electric arc melting furnace (with a chamber volume of 18 L) under different absolute pressures and various H_2_ fractions,^11,12^ as summarized in Table II. To evaluate the effects of the total pressure on the reduction kinetics, 15 g hematite was reduced with a hydrogen plasma created under an atmosphere containing 10% H_2_ with absolute pressures of 450 and 900 mbar (Table II). The number of moles of H_2_ and Ar inserted in the furnace for these two experimental conditions (assuming that they behave as ideal gases before the reduction) is $n_{{{\text{H}}_{2} }}^{450} = 0.033\;{\text{moles}},\;n_{{{\text{Ar}}}}^{450} = 0.293\;{\text{moles}}$ and $n_{{{\text{H}}_{2} }}^{900} = 0.065\;{\text{moles}},n_{{{\text{Ar}}}}^{900} = 0.586\;{\text{moles}}$, respectively. The hematite portion was placed on the water-cooled Cu hearth of the furnace (anode), lying immediately underneath the tungsten electrode (cathode). The arc was ignited between the cathode and anode with a current of 200 A. The input oxide was simultaneously melted and reduced for 2 min. After switching off the arc, the sample rapidly solidified inside the furnace because of the cooling imposed by the Cu hearth. A second portion of 15 g-hematite was reduced for 10 min. In this case, the chamber of the furnace was replenished with fresh Ar-10% H_2_ gas mixtures after 2 min of exposure to the reducing plasma. For the continuation of the redox chemical reactions, the appropriate stoichiometry of gaseous reagents in the system was guaranteed by replenishing the atmosphere of the furnace with fresh Ar-10%H_2_ mixtures after the completion of every minute of melting and reduction.^11^ This protocol was performed eight times, leading to a total of 10 min of reduction.

**Table I:** Chemical composition of hematite utilized in this work, given in wt.%

Fe	Si	Al	Mn	Ti	Ca	Mg	S	P	O
Bal	0.10	0.087	0.09	0.04	0.025	0.048	0.145	0.014	28.90

**Table II:** Pressure conditions of the experimental reduction of hematite via HyPSR

Condition	Total pressure (mbar)	Pressure of H_2_ (mbar)	Pressure of Ar (mbar)	Reducing time, min
1	450	45 (10%)	405 (90%)	2, 10
2	900	90 (10%)	810 (90%)	2, 10
3	450	90 (20%)	360 (80%)	2, 5

To investigate the effects of the H_2_ partial pressure on the reduction kinetics, an 18-g hematite sample was melted and reduced in the same electric arc melting furnace under an atmosphere of Ar-20% H_2_ at 450 mbar (the number of moles inside the chamber of the furnace before reduction is as follows: $n_{{{\text{H}}_{2} }}^{450} = 0.065\;{\text{moles}},n_{{{\text{Ar}}}}^{450} = 0.260\;{\text{moles}}$), Table II. Two samples were reduced for 2 and 5 min, respectively, adopting the same protocol used before. The metallization degree was quantified for all solidified samples.

### Characterization of the Solidified Samples

The solidified samples were hammered to magnetically separate the millimeter-sized Fe portions from the remaining unreduced domains. The oxide portions were further powdered to an average particle size of 90 μm and submitted to X-ray diffraction (XRD) analysis using a D8 Advance A25-X1 diffractometer, equipped with a cobalt Kα X-ray source, operated at 35 kV, 40 mA. The diffraction data were evaluated via Rietveld refinement to quantify the weight fraction of constituents present in the unreduced oxide, including micron-sized Fe islands entrapped in the oxide domains because of the fast solidification conditions imposed by the water-cooled Cu hearth. The metallization degree was calculated by the ratio between the mass of metallic Fe (both micro- and millimeter-sized domains) and the total mass of the corresponding solidified sample.^11^ The total content of O and Fe in each sample was inferred from the stoichiometry of the constituents found in each sample (viz., Fe, FeO, Fe_3_O_4_, FeSiO_4_) and used to evaluate the corresponding O and Fe losses during the reduction via either reduction with hydrogen plasma species or evaporation.^12^

## Results and Discussion

### The Impact of Pressure on HyPSR

**Fig. 3:**
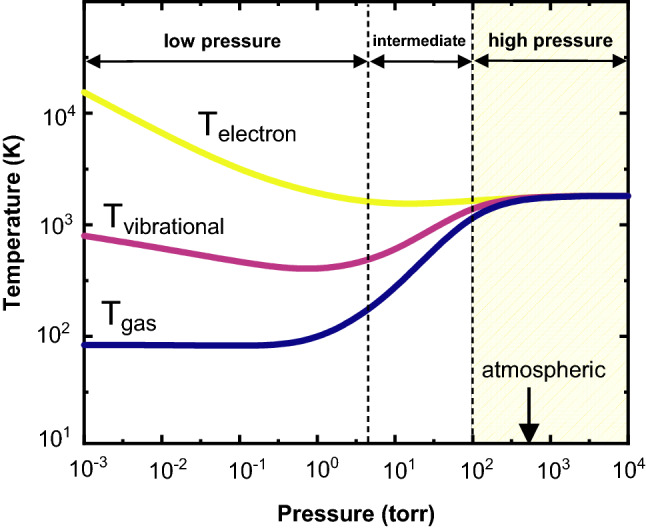
Typical temperature of heavy particles (neutral and excited) and electrons in non-thermal and thermal plasmas, as a function of pressure. Thermal plasmas ignited at high pressures, as highlighted by the yellow frame, are preferred in large-scale reactors for industrial application purposes. T_gas_, T_v_ and T_e_ stand for the temperatures of gaseous molecules, vibrational states and electrons, respectively. Adapted from Ref. 33 (Color figure online).

Hydrogen-containing plasmas created in low-pressure chambers can contain substantial populations of high-energy hydrogen species such as H atoms,^26^ whose reducing potential is higher than that of the H_2_ molecular variant.^27^ Under such conditions, these particles can be created at relatively low temperatures (e.g., 200°C), a fact that permits one to use low-temperature plasmas for chemical vapor deposition,^28^ and solid-state reduction of different oxides,^29^ including iron ores.^30,31^ These plasmas are called non-thermal plasmas as the electrons and heavy particles assume different temperatures, Fig. 3.

However, in industrial-scale smelting set-ups such as electric arc furnaces (EAF), DC or AC-generated thermal plasmas ignited under atmospheric pressures are more attractive,^32^ as their high temperatures (typically ranging from 2000 K to 20,000 K) enable processing/smelting hundreds of tons of input material in a single batch process (e.g., an EAF can process 100 tons of steel scrap in a single batch process). Thermal plasmas are those in which the temperature of electrons is fairly the same as that of heavy ions,^33^ as shown in Fig. 3. Under such circumstances, the different plasma species are assumed to exist under local thermodynamic equilibrium (LTE).

For the HyPSR technology, modified EAF to support small fractions of hydrogen (e.g., 5–10%) inside its volume would make use of thermal hydrogen plasmas to simultaneously melt and reduce the input iron ores, thus exploiting the high diffusivity in the liquid to conduct the redox chemical reactions at enhanced kinetics.^11^ Similarly important in HyPSR is to ensure a high density of highly energetic hydrogen plasma species (e.g., H atoms) close to the reaction interface existing between the molten material and the plasma arc. As metastable hydrogen plasma species are more reactive than the molecular variant (H_2_), they have potential to enhance the reduction kinetics of iron ores by an order of magnitude,^23,33,34^ also equipping them with an exothermic net energy balance.^27^ The latter fact enables thermal and electrical energy savings. It is also important to recall that the solid-state reduction of wüstite (FeO) with molecular hydrogen H_2_ is endothermic, thus requiring an external energy supply to compensate for the temperature drop during HyDR.^10^

**Fig. 4:**
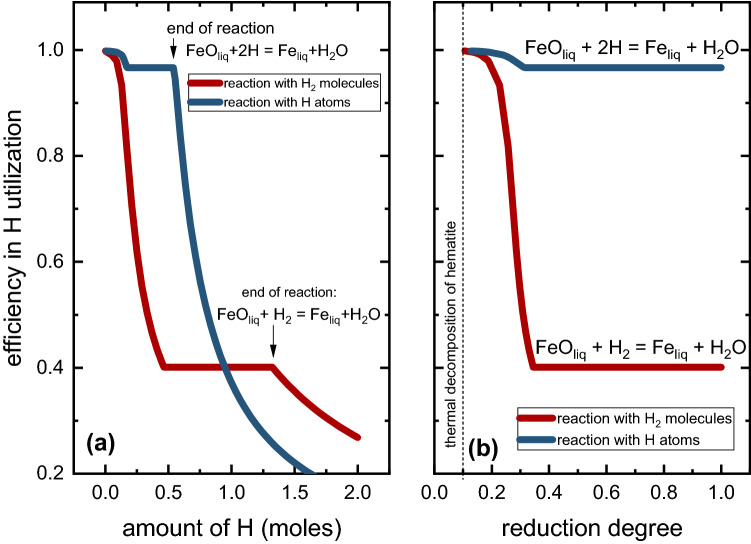
Efficiency in hydrogen utilization over reduction of a molten iron oxide (Fe-29 wt.% O, namely, hematite when in solid state) exposed to deliberatively increasing amounts of H_2_ molecules and H atoms, at 2000°C and 1 bar. The efficiency in hydrogen utilization is plotted as a function of (a) the amount of H provided to the system, given in moles; (b) the reduction degree, calculated based on the mass loss of O. The vertical dashed line shows the reduction degree due to the thermal decomposition of hematite.

Another advantage of conducting the reduction of iron oxides with hydrogen plasma species as reducing agents is the high efficiency in (costly) hydrogen utilization.^22,23,25^ This aspect in the ironmaking sector is of utmost importance to render green steel production economically viable, with minimum efforts in recycling and purifying it from the off-gas by-products.^12^ We calculated the theoretical efficiency in hydrogen consumption under equilibrium conditions during the reduction of hematite at 2000°C using ThermoCalc coupled with the TCOX10 and SUBB5 databases ("Hydrogen Plasma Smelting Reduction of Hematite" section). For this purpose, a 15 g liquid oxide (Fe-28.9 wt.% O) was exposed to increasing amounts of a gas mixture of Ar-10% H_2_, under atmospheric pressure conditions. The hydrogen species were chosen to be in molecular (H_2_) and atomic (H) forms in two separate sets of calculations. The results obtained are displayed in Fig. 4 as a function of the number of hydrogen atoms provided to react with the liquid ore (Fig. 4a) and the reduction degree (Fig. 4b). The reduction degree was calculated based on the mass of oxygen removed from the oxide liquid (Fig. 4b). The vertical line in Fig. 4b highlights the reduction degree of 10% caused by the thermal decomposition of hematite when heated at 2000°C. At this temperature, the system is found in the stability field of liquid oxide plus molecular oxygen (O_2_) according to the Fe-O phase diagram. Thus, under equilibrium conditions, up to 10% reduction, no oxygen is extracted from the melt via reaction with hydrogen species, but rather evaporated in the form of O_2_.

**Fig. 5:**
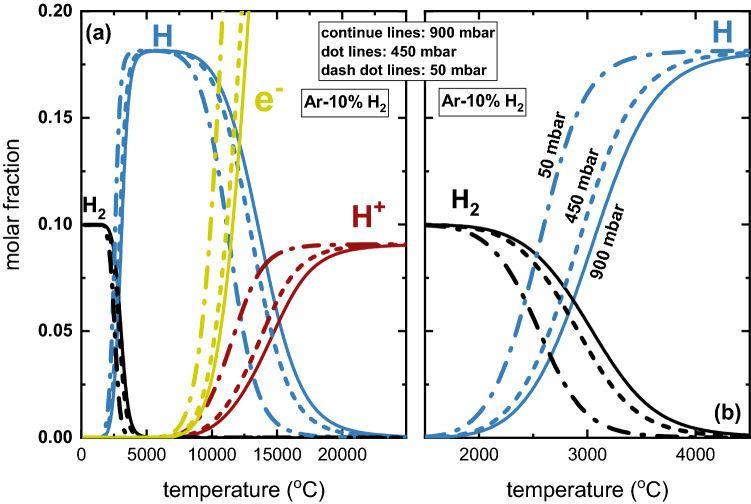
Equilibrium calculations for gas mixtures of Ar-10% H_2_ under different absolute pressures (50 mbar, 450 mbar and 900 mbar) within the temperature range varying from 0°C to 25,000°C, (a). The continuous, dotted and dashed lines show the equilibrium compositions at 900 mbar, 450 mbar and 50 mbar, respectively. (b) Closer view of the equilibrium compositions within the temperature range from 1500°C to 4500°C. For the sake of simplicity, only hydrogen species (H_2_ molecules, H atoms, H^+^ ions) and electrons (e^−^) are depicted.

Figure 4 shows that the reaction with H_2_ proceeds relatively efficiently within the initial reduction interval from 10% to 38%. However, the consumption of hydrogen drops to approximately 40% with the progress of the reaction, i.e., during the reduction of the liquid iron oxide displaying a stoichiometry of Fe^+2^O^−2^. This result agrees well with experimental observations reported in the literature, especially for HyPSR devices.^13,22,23^ This also suggests that the major active reducing agent in the reactors employed in Refs. 13, 22, and 23 was hydrogen molecules (H_2_). Conversely, Fig. 4 also shows that the entire course of the reduction can be made much more efficient in terms of hydrogen consumption when only H atoms are used as a reducing agent, especially during the reduction of the oxidic liquid composed of Fe^+2^O^−2^, with an efficiency of 98%.^23^ This means that, in principle, the higher the concentration of H atoms near the reaction interface in HyPSR, the higher is their efficiency in participation in the redox chemical reactions and the lesser the amount of non-consumed (lost) hydrogen that needs to be collected in post-processing purification and recycling. Therefore, it is highly desired to identify strategies to increase the population of H particles (i.e., increase the rate of dissociation events of H_2_ into H) in the regions inside hydrogen thermal plasmas that are close to the reaction interface. Increasing the temperature to fully transform the H_2_ molecules into H atoms or H^+^ ions would not be an interesting solution as the most reactive species, such as the proton H^+^, are expected to exist at temperatures well above the boiling point of wüstite (3114°C) or iron (2862°C), as shown in Fig. 5. In this context, the role of total pressure or fraction of hydrogen inside the reactor could positively contribute to enhancing the density of hydrogen plasma species at temperatures below the boiling point of iron oxides or iron.

**Fig. 6:**
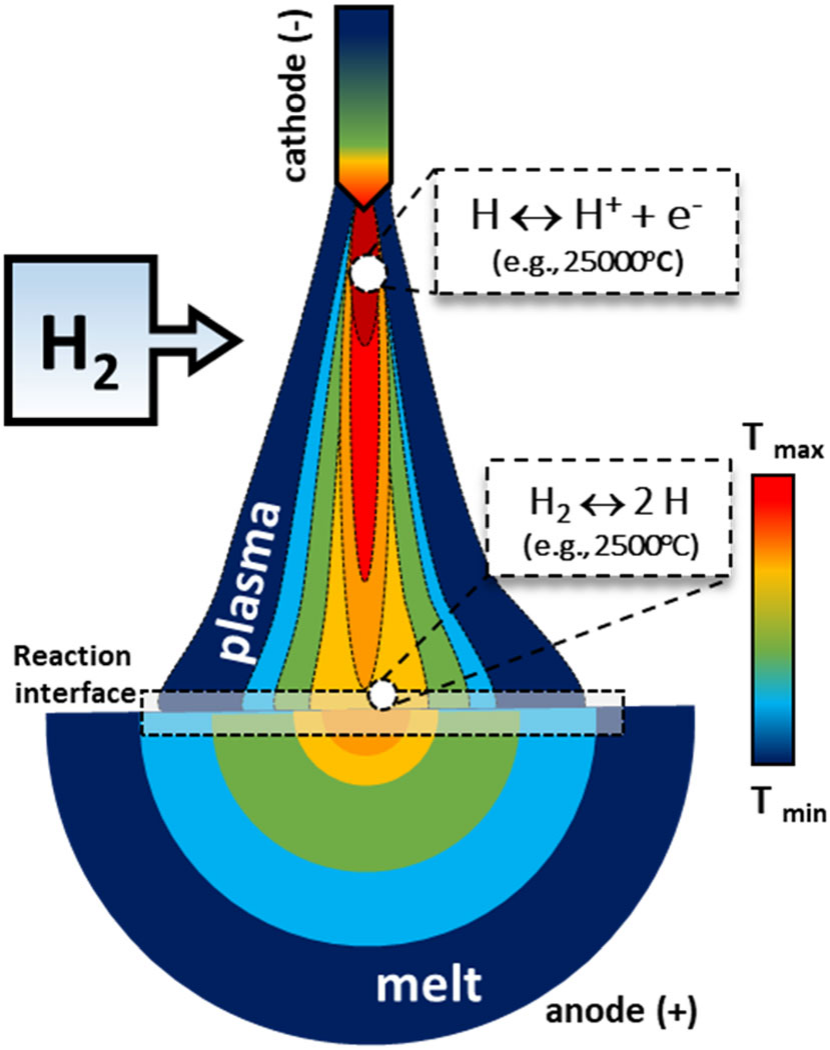
Schematic representation of the temperature distributions inside a hydrogen-containing electric arc and a molten iron-based material, constructed based on Refs. 22, 35, and 36. The reaction interface between the arc and melt is highlighted by the dashed frame. Possible dissociation reactions involving H species are also shown in particular regions of the arc.

To showcase the impact of the total pressure on the density of hydrogen plasma species, we calculated the equilibrium molar fraction of H_2_, H, H^+^ and electron (e^−^) for a gas mixture of Ar-10%H_2_ under different pressures, as shown in Fig. 5. For the sake of clarity, the Ar species were omitted in this figure. Figure 5 reveals that H_2_ molecules start dissociating into H atoms (H_2_ ↔ 2 H) at ~ 1800°C for an atmosphere at 900 mbar. Conversely, for the same pressure magnitude, H^+^ can only be expected to exist at temperatures  > 5500°C. This result means that H^+^ would only be present at the hottest positions of the arc, as the domains nearby the cathode, which, in turn, are far from the reaction zone, as schematically displayed in Fig. 6.

When evaluating the temperature profile of hydrogen-containing plasma arcs either determined through modelling for welding setups^35,36^ or estimated for HyPSR purposes^22^ (as schematically depicted in Fig. 6), one can expect that the reaction interface can display temperature values varying from 2000°C to 2500°C (Fig. 6), which are below the boiling point of the materials of interest (FeO, Fe). Also, the product liquid iron formed through HyPSR sinks to the bottom of the melt (cold regions of the bath, Fig. 6) because of a higher density than that of the oxide counterparts. This minimizes the possibility of iron evaporation by being less exposed to the highest temperatures near the reaction interface.

In light of these observations, one might consider the possibility of exploiting moderate (50–100 mbar) and intermediate (450 mbar) total pressures (Fig. 5) in HyPSR to increase the molar fraction of plasma species at relatively lower temperatures and equip the redox chemical reactions during HyPSR with enhanced kinetics and efficiency in hydrogen utilization. This seems to be of particular importance when one aims at increasing the number of H atoms near the reaction interface between the arc and the melt, enabling the reduction to proceed under the following reaction: FeO + 2H → Fe + H_2_O (e.g., at 2500°C). Figure 5 shows that a decrease in the total pressure to 450 mbar (a value that is still in the regime of high-pressure thermal plasmas, Fig. 3) increases the fraction of H atoms at 2500°C (0.033) by approximately 40% in relation to that found at 900 mbar (0.024). At a lower pressure of 50 mbar, the impact is even higher and 2.3 times more H atoms (molar fraction of 0.08) exist at 2500°C compared with the molar fraction found at 900 mbar.

### Impact of the Hydrogen Fraction on HyPSR

**Fig. 7:**
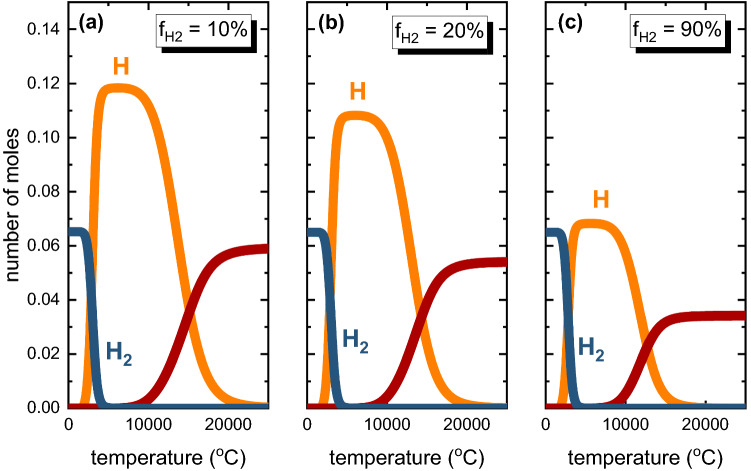
Equilibrium compositions for gas mixtures of Ar and H_2_ containing H_2_ molar fractions (f_H2_) of (a) 0.10, (b) 0.20 and (c) 0.90. In all cases, the calculations were conducted for a total pressure of 900 mbar. For the sake of simplicity, only H species are shown in this figure.

From a thermodynamic perspective, increasing the molar fraction of hydrogen molecules in a gas mixture of Ar and H_2_ leads to lower amounts of H atoms existing in equilibrium with Ar and H_2_, as shown in Fig. 7 (for the sake of clarity, only the H species are shown in this figure). The boundary conditions for the equilibrium shown in Fig. 7 were a total pressure of 900 mbar and the same initial number of moles of H_2_ molecules (0.065 mol). The number of moles of Ar varied accordingly to provide different initial H_2_ molar fractions (f_H2_) of 0.1, 0.2 and 0.9, respectively shown in Figs. 7a–c.

Experimentally, Kamyia et al.^23^ and others^22,25^ have also observed that an increase in the H_2_ concentration in the input gas utilized for the reduction of FeO-containing melts reduces both the reduction kinetics rates and the hydrogen utilization efficiency. In this context, the hypothesis is that by increasing the faction of H_2_ within the plasma environment, the mutual recombination events between H species into H_2_ molecules can be favored (H_2_ ← 2H),^22^ decreasing the concentration of H atoms available to participate in the reduction reactions, thus reducing the hydrogen utilization efficiency (H_2_ molecules render the redox reactions less efficient as shown in Fig. 4). Handling deliberative large quantities of hydrogen inside plasma reactors is not only kinetically and thermodynamically disadvantageous, but also not attractive from a safety perspective, as one aims at operating such reactors below the flammability risks.

### Experimental Approaches in HyPSR

**Fig. 8:**
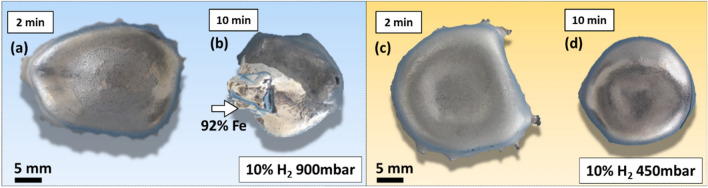
Top view of iron ores reduced via HyPSR using a gas mixture of Ar-10% H_2_. Samples reduced at a total pressure of 900 mbar for (a) 2 min, (b) 10 min (the white arrow highlights the major portion of metallic Fe). Samples reduced at a total pressure of 450 mbar for (c) 2 min, (d) 10 min.

To validate the theoretical insights achieved through the thermodynamic simulations, we conducted a series of melting and reduction experiments via HyPSR under different conditions of total and H_2_ partial pressures. Such experimental validation of the impact of pressure on the reduction kinetics is important to shed light on the practical feasibility of adjusting this parameter in hydrogen plasma reactors. In the first set of experiments, 15 g hematite was reduced for 2 min and 10 min under 900 mbar using a gas mixture of Ar-10%H_2_. The top view of the obtained samples is shown in Fig. 8a and b. The white arrow depicted in Fig. 8b highlights the major portion of metallic Fe formed after 10 min of exposure to hydrogen-containing plasma. For the second set of experiments, all parameters were kept identical except the total pressure, which was reduced to 450 mbar. The samples reduced for 2 min and 10 min are shown in Fig. 8c and d, respectively.

**Fig. 9:**
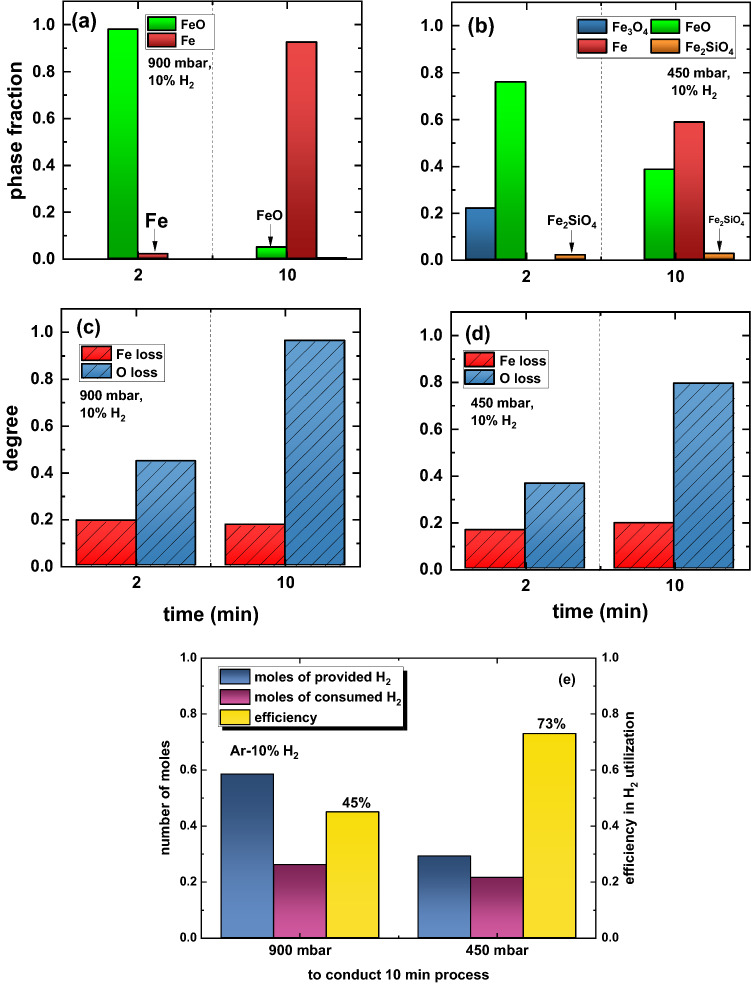
Phase fractions of the solidified samples after reduction via HyPSR using a gas mixture of Ar-10%H_2_ and under a total pressure of (a) 900 mbar and (b) 450 mbar. Corresponding Fe and O losses for the same samples reduced under a total pressure of (c) 900 mbar and (d) 450 mbar. (e) Quantities of hydrogen provided to the furnace and consumed over the course of 10 min reduction, conducted at a total pressure of 900 and 450 mbar. The efficiency in H_2_ utilization, i.e., the ratio between the consumed and provided hydrogen, is also shown in this figure (Color figure online).

The phase fractions for the samples reduced under 900 mbar and 450 mbar are shown in Fig. 9a and b, respectively. The phase quantification was indispensable to infer the absolute amount of O and Fe present in each sample and partitioned among different constituents, mostly represented by wüstite (FeO), magnetite (Fe_3_O_4_) and iron (Fe).^11,12^ Traces of fayalite (Fe_2_SiO_4_) are also found in the solidified samples processed under 450 mbar. This silicate is a product of minor side chemical reactions between iron oxides and the gangue-related impurities, especially SiO_2_, that are originally contained in hematite used in this work (Table I). Once the absolute mass of O and Fe in each sample is known, we can calculate the O and Fe losses, as given in Fig. 9c and d, respectively. The O loss translates the removal of oxygen (reduction degree) from the melt either via thermal decomposition of the material or through chemical reactions with hydrogen species stemming from the arc. After 10 min of reduction at 900 mbar, nearly 95% O loss is achieved whereas 80% is obtained for the same exposure time to hydrogen-containing reducing plasma at 450 mbar. The Fe loss can represent the amount of this element evaporated over the process, or the small portions of material that might get eventually spattered because of the initial (violent) ignition of the arc.^12,37^ For both experimental conditions, about 20% of all Fe from the input hematite (10.67 g) was lost, a number that translates into an absolute mass of approximately 2 g. When evaluating the equilibrium conditions for the chemical reactions between a molten iron ore and molecular hydrogen at 900 mbar in Fig. 4, we found that the equilibrium composition of the resulting gas contains 2.41 g of vapor Fe. This result is in good agreement with the experimental findings, thus suggesting that Fe evaporation is inevitable over the reduction of molten iron ores conducted at high pressures.

These observations reveal a strong influence of the total pressure of the system with identical initial gas mixtures containing Ar-10% H_2_ on reduction kinetics and gas utilization efficiency. At 900 mbar, hydrogen-containing plasma promotes a substantial production of Fe (92% metallization on a weight basis) after 10 min process. Conversely, the total pressure of 450 mbar leads to a lower metallization of 60%. Halving the total pressure of the system to 450 mbar while keeping its volume unaltered (i.e., the volume of the reactor chamber, 18 L, is a fixed parameter in the experiments) also implies halving the number of moles of hydrogen gas inserted in the furnace for the reaction, as shown in Fig. 9e by the blue columns. Once the availability of hydrogen species to serve as a reducing agent at 450 mbar is scarcer than that of experiments performed at 900 mbar, the conversion kinetics proceeds more sluggishly. However, the efficiency in H_2_ utilization increases from 45% for the experiments conducted at 900 mbar (a fact that suggests that H_2_ molecules were the most prominent reducing agent over the course of the process, Fig. 4) to 73% at 450 mbar. At this point, it is important to recall that 10 min reduction conducted at an absolute pressure of 450 mbar promoted about  ~ 80% oxygen loss and not full conversion into iron (Fig. 9d). The efficiency in H_2_ utilization to achieve complete reduction at 450 mbar was monitored in another set of experiments. The obtained results are documented in the Supplementary Material and reveal that full conversion into iron is obtained after 13 min of exposure to reducing hydrogen plasma (at 450 mbar) with an efficiency in H_2_ utilization of 67%. This result evidences that the concentration of more reactive H plasma species, other than H_2_ molecules, can be enhanced in hydrogen-containing electric arcs when the total pressure of the system is reduced. Such an observation agrees with the theoretical findings reported in Figs. 4 and 5, thus helping to save costly hydrogen in HyPSR reactors.

**Fig. 10:**
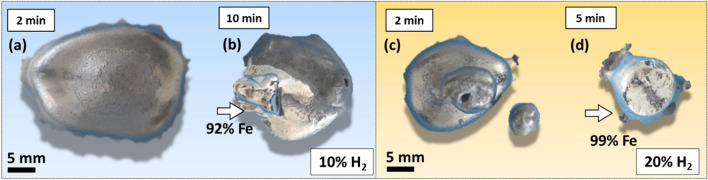
Top view of iron ores reduced via HyPSR. Samples reduced with an H_2_ molar fraction of 0.10 for (a) 2 min, (b) 10 min. Samples reduced with an H_2_ molar fraction of 0.20 for (c) 2 min, (d) 5 min (the total pressure is 450 mbar).

To evaluate the impact of the hydrogen fraction of the input gas on the reduction kinetics, hematite pieces were reduced for 2 min and 5 min under an atmosphere containing an H_2_ fraction of 0.20 (see "Characterization of the Solidified Samples" section). The reduced samples are shown in Fig. 10c and d, respectively, in compared with the samples reduced at 10% H_2_ for 2 and 10 min which were already shown in Fig. 7a and b. The white arrows in Figs. 10b and d indicate the portions of fully reduced metal.

**Fig. 11:**
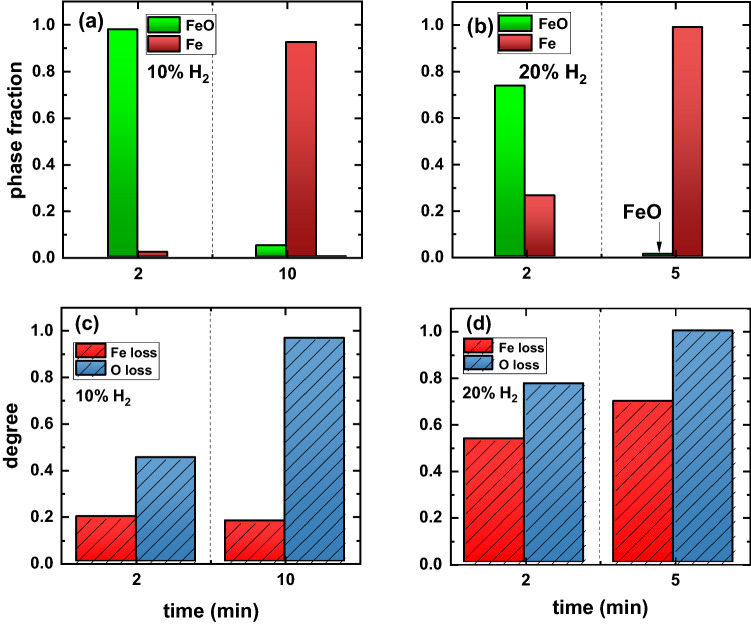
Phase fractions of the solidified samples after reduction via HyPSR using a gas mixture of Ar-H_2_ containing a H_2_ molar fraction of (a) 0.10 and (b) 0.20. Corresponding Fe and O losses for the same samples reduced under atmospheres containing (c) 10% H_2_ and (d) 20% H_2_.

The corresponding phase fraction evolution for these samples is documented in Fig. 11a and b. The most prominent phases in the solidified samples are wüstite (FeO) and iron (Fe). The O and Fe losses are shown in Fig. 11c and d. The results reported in Fig. 11 reveal that by doubling the H_2_ percentage to 20%, the reduction kinetics proceeds faster than that observed for 10% H_2_ so that a metallization degree of approximately 30% is achieved with only 2 min of exposure to the hydrogen-containing plasma with 20% H_2_ gas input. However, the process is accompanied by staggering evaporation of Fe and about half of its total amount originally contained in hematite is lost via gas phase within the initial 2 min, Fig. 11d. After 5 min of reduction, the sample is almost pure metallic Fe (Fig. 11b), but about 70% of the total Fe was evaporated (Fig. 11d). This observation suggests that the temperatures associated with the employed arcs can be much higher than those ignited under an atmosphere containing an H_2_ percentage of 10%.

Modelling studies reported that hydrogen additions to Ar arcs increase their thermal conductivity and voltage drop, thus increasing the arc power.^35,38,39^ Because the thermal conductivity of hydrogen is higher than that of Ar, the resulting hydrogen-containing arcs are narrower and display higher temperature distributions, especially near the reaction interface.^35,36^ These facts permit deep penetration inside the molten material making its temperature distributions below the arc exceed the boiling temperatures of Fe or FeO, thus causing unwanted evaporation of Fe. Our preliminary theoretical and experimental evaluation aims at shedding light on the huge avenue of opportunities to further investigate the impact of atmospheres regarding H_2_ fraction and total pressure, to be utilized in HyPSR. Comprehensive understanding of the physical, chemical, thermal and associated engineering aspects of hydrogen-containing electric arcs, namely, temperature distribution and density of H atoms existing under different conditions of the process—and how to tune them is an important field to be further developed to maximize the efficiency of hydrogen utilization in electric arc furnaces and simultaneously inhibit the evaporation of desired elements.

## Summary and Conclusion

In the context of the solid-state HyDR, the partial pressure of gaseous hydrogen (p_(H2)_/(p_(H2)_ + p_(H2O)_)) exerts a more prominent impact on the reduction when compared with the effects of the absolute pressure, as the former influences the driving force of the redox chemical reactions. From a kinetics perspective, an increase in the H_2_ partial pressure also renders more moles of H_2_ molecules within a confined volume of a reactor, thus enhancing the probability of gas molecule dissociation events and associated collisions with iron oxides surface.

The absolute gas pressure and H_2_ fractions have a significant impact on the HyPSR process, as they directly affect the thermodynamic and kinetic aspects of the dissociation events of H_2_ molecules into metastable H plasma species, such as H and H^+^. In HyPSR reactors, it is highly desired to increase the density of H atoms close to the reaction interface between the plasma arc and the molten material. This is because H atoms are more reactive than H_2_ molecules, promoting better utilization of hydrogen.

- Our thermodynamic modelling shows that a decrease in the total pressure of the Ar-H_2_ atmosphere enhances the density of highly energetic H atoms at temperatures below the boiling point of Fe oxides and Fe, especially at 2500°C, which is a typical temperature found near the reaction interface. Validation experiments provided good insights into this direction and demonstrated that decreasing the total pressure of the system from 900 mbar to 450 mbar permits an H_2_ utilization of 73%, suggesting also that the population of H atoms could be increased near the reaction interface. Besides, Fe evaporation is inevitable because of thermodynamic equilibrium conditions and its losses were kept at the minimum value of 20%.
- An increase in the H_2_ concentration in the Ar-H_2_ gas mixture decreases the dissociation rates of H_2_ molecules into H atoms, as reported in the literature and confirmed by our thermodynamic calculations. With lower quantities of metastable H to participate in the reaction, lower H_2_ utilization is expected. Besides, our experiments conducted for an H_2_ molar fraction of 0.2 resulted in pronounced evaporation of material, especially of the product Fe. This could be attributed to the fact that additions of H_2_ to argon plasmas increase the temperature of the resulting arc, enhancing the chances of evaporation of the processed material.
- This works aims at providing the first insights into the role of pressure in the reduction of Fe ores with hydrogen, specifically in HyPSR. The observations suggest that both lower fraction of H_2_ in the input gas and absolute pressures are desired conditions for the reduction of Fe ores with optimized utilization of costly hydrogen. With these findings we aim to open the path for further fundamental research and technological development in hydrogen plasma smelting reduction approaches, an essential route to electrifying the ironmaking sector with sustainable and economic practices.

## Supplementary Information

Below is the link to the electronic supplementary material.

Supplementary file1 (PDF 76 KB)
