# Supplementary material for: Fundamentals of Green Steel Production: On the Role of Gas Pressure During Hydrogen Reduction of Iron Ores
Source: JOM (1989). 2023 May 1;75(7):2274–86. doi: 10.1007/s11837-023-05829-z (PMC10282048; doi:10.1007/s11837-023-05829-z)
Supplement: Supplementary file 1 — Supplementary file1 (PDF 76 KB) [file 11837_2023_5829_MOESM1_ESM.pdf]

Supplementary Material

Fundamentals of green steel production: On the role of gas pressure during hydrogen reduction of iron ores

I. R. Souza Filho[1,*], Y. Ma[1], D. Raabe[1], H. Springer[1,2]

[1] *Max-Planck-Institut für Eisenforschung, 40237 Düsseldorf, Germany*
[2] *Metallic Composites, RWTH Aachen University, 52072 Aachen, Germany*

* *corresponding author:* i.souza@mpie.de

Full conversion of hematite into iron through HyPSR at 450 mbar

Additional HyPSR experiments were conducted at an absolute pressure of 450 mbar adopting the same experimental conditions reported in Section 2.2 of the manuscript. The aim of this additional set of experiments is to monitor the reduction kinetics during the full transformation of 15-g hematite into metallic iron through HyPSR at 450 mbar. The obtained results are as shown in Supplementary Figure 1 (a) in terms of oxygen loss. This figure also shows the reduction kinetics of the counterpart experiments performed at 900 mbar (already documented in Fig. 9 (c) of the manuscript) for the sake of comparison. Supplementary Figure 1 (a) reveals that full conversion into iron is achieved with 13 min of exposure to the reducing plasma at an absolute pressure of 450 mbar.

The quantities of hydrogen inserted into the furnace and the ones consumed during the process are reported in Supplementary Figure 1 (b) together with the corresponding efficiency in H_2 utilization for the experiments conducted at 450 and 900 mbar. This figure shows that full conversion into iron though HyPSR at 450 mbar proceeds with an efficiency in H_2 utilization of 67%, whereas only 45% of all H_2 provided to the system is utilized when the reduction process is conducted at 900 mbar.

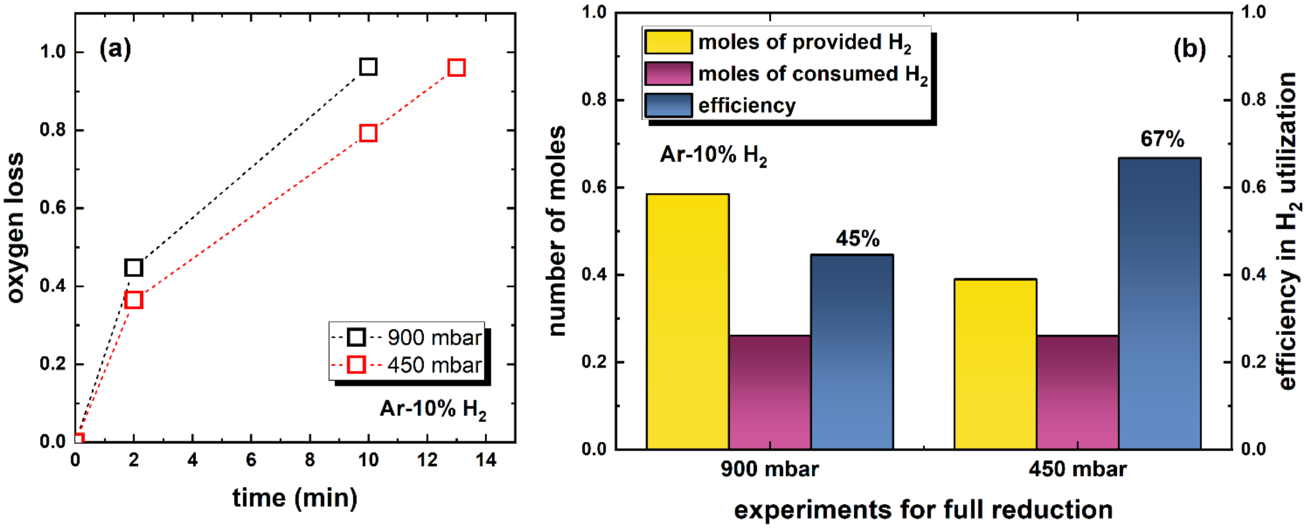

Supplementary Figure 1. Hydrogen plasma smelting reduction of hematite (HyPSR). **(a)** Reduction kinetics, expressed in terms of oxygen loss, for experiments conducted at absolute pressures of 900 and 450 mbar. **(b)** Quantities of hydrogen provided to the furnace and consumed to reach complete reduction into metallic iron, at a total pressure of 900 and 450 mbar. The efficiency in H_2 utilization, i.e. the ratio between the consumed and provided hydrogen, is also shown in this figure.
